# Comparison of clinical outcomes between unilateral biportal endoscopic transforaminal lumbar interbody fusion and oblique lumbar interbody fusion with posterior percutaneous screw fixation in patients with lumbar spinal canal stenosis

**DOI:** 10.3389/fsurg.2026.1789093

**Published:** 2026-03-30

**Authors:** Ye-Hui Wang, Xiang Gao, You-Peng Hu, Guo-Sheng Tang, Wei Cui, Shi-Peng Yang, Si-Mao Song, Wei Hou, Xuan-Geng Deng

**Affiliations:** 1 Department of Spine and Neurosurgery, Sichuan Province Orthopedic Hospital, Chengdu, Sichuan, China; 2 The Second Medical Center & National Clinical Research Center for Geriatric Diseases, Chinese PLA General Hospital, Beijing, China; 3 Department of Spine, Mianyang Orthopedic Hospital, Mianyang, Sichuan, China

**Keywords:** lumbar spinal canal stenosis, minimally invasive surgery, oblique lumbar interbody fusion, spine surgery, unilateral biportal endoscopy

## Abstract

**Purpose:**

This study aims to evaluate the clinical outcomes of unilateral biportal endoscopic transforaminal lumbar interbody fusion (ULIF) and oblique lumbar interbody fusion with posterior percutaneous screw fixation (OLIF-PF) in the treatment of lumbar spinal canal stenosis (LSCS).

**Methods:**

Data from December 2019 to January 2022 were analyzed. 54 patients in the ULIF group and 42 patients in the OLIF-PF group completed a minimum of 1 year follow-up. The demographic data, clinical indicators, laboratory indicators, radiological results, and complications were assessed and compared between the two groups.

**Results:**

There was no significant difference in the preoperative baseline between the two groups. The ULIF group experienced shorter operation time and more blood loss compared with the OLIF-PF group (*P* < 0.05). The VAS for back pain and ODI score at 1 day postoperatively were lower in the OLIF-PF group than in the ULIF group. At the last follow-up, the VAS for back pain was lower in the ULIF group than in the OLIF-PF group (*P* < 0.05). However, the VAS for leg pain was lower in the ULIF group than in the OLIF-PF group at 1 day postoperatively, and there were no significant differences at other follow-up times (*P* < 0.05). There was no significant difference between the two groups in the modified MacNab score at last follow-up (90.74% vs. 90.48%, *P* > 0.05). And there was no significant difference between the two groups in CPR and ESR (*P* > 0.05). The improvements of DH, LL, and SA were superior in the OLIF-PF group than in the ULIF group at 3 days postoperatively and maintained to the last follow-up (*P* < 0.05).

**Conclusions:**

Both ULIF and OLIF-PF showed favorable mid-term outcomes in the treatment of LSCS. Both surgical methods significantly improved pain symptoms and lumbar sagittal balance. Long-term follow-up and larger clinical studies are needed to confirm this result.

## Introduction

Lumbar interbody fusion (LIF) is a widely recognized and effective surgical technique used to treat lumbar spinal canal stenosis (LSCS) (1). LIF surgery can achieve complete decompression of the spinal canal and reconstruction of spinal function, greatly improving the stability of the spine, and has been proved to have satisfactory efficacy and safety (2, 3). Both posterior lumbar interbody fusion (PLIF) and transforaminal lumbar interbody fusion (TLIF) are frequently employed, offering benefits such as segmental fusion promotion, nerve root decompression, pain relief, and restoration of the spine's natural alignment, which have been well-documented in clinical practice (4). Nevertheless, the adverse effects associated with traditional open LIF procedures, such as extensive muscle dissection and soft tissue damage, remain a concern, leading to additional complications (5). In response to these challenges, spine surgeons are increasingly focusing on minimally invasive techniques, including the anterior (ALIF), lateral (LLIF), and oblique (OLIF) approaches, which aim to reduce surgical invasiveness. Despite its benefits, ALIF is frequently associated with complications like abdominal vascular injury (6), while LLIF can lead to damage to the lumbar plexus or psoas major muscle (7). OLIF, on the other hand, utilizes the natural space between the retroperitoneal fat and the psoas major muscle to access the target segment (8), allowing for indirect decompression of the spinal canal and reconstruction of the vertebral alignment by restoring intervertebral height, which can better overcome the complications associated with ALIF and LLIF (9). OLIF performed without screw fixation has been associated with an elevated risk of cage subsidence (1), with some studies reporting a rate as high as 30%, which may lead to decompression failure or even secondary surgery (10). For reducing the incidence of cage subsidence, OLIF combined with posterior percutaneous screw fixation was introduced. Studies have shown that in the treatment of LSCS, OLIF-PF demonstrated better repositioning and long-term maintenance of efficacy (11, 12). Furthermore, the advancement of percutaneous endoscopic spinal techniques has facilitated the use of endoscopic assistance in lumbar fusion procedures. ULIF is an emerging surgical method that is getting increased attention from spinal surgeons (13). It combines the benefits of both open surgery and minimally invasive approaches, offering reduced tissue damage, less blood loss, and quicker recovery, while still maintaining the advantages of open surgery such as a broader operating field and flexibility in procedural range (14). ULIF has demonstrated satisfactory clinical outcomes and safety in the treatment of LSCS (15).

Although ULIF and OLIF-PF have different surgical approaches and surgical procedures, both surgical methods can improve LSCS. However, comparative studies assessing the clinical efficacy of ULIF vs. OLIF-PF are limited. Therefore, we conducted this retrospective study to evaluate and compare the short-term clinical and radiographic outcomes of these two approaches in treating LSCS.

## Methods

### Study population

After receiving approval from the institutional review board, a retrospective study was conducted on consecutive patients who underwent either ULIF or OLIF-PF between December 2019 and January 2022 at the spine center of our institution. Given the retrospective nature of the study and the anonymous analysis of data, informed consent was not required from participants. The selection of the surgical procedure was made collaboratively between the patient and the senior spine surgeon. Although the surgical approach is co-decided by the patient and a senior spine surgeon, the ULIF technique is the primary recommendation and choice for patients with severe central spinal canal stenosis (MRI sagittal canal diameter <10 mm) (16). This recommendation creates some variation in surgical selection, but it does not significantly impact the clinical outcomes comparison between the two procedures for LSCS.

### Inclusion and exclusion criteria

The inclusion criteria were as follows: (1) patients diagnosed with lumbar spinal stenosis; (2) no significant improvement in more than three months of systematic conservative treatment; (3) single-segmental lesion. The exclusion criteria were: (1) prior lumbar surgery; (2) severe osteoporosis; (3) presence of lumbar tumors, lumbar tuberculosis, or other infectious conditions.

### Surgical procedure

**Figure 1:**
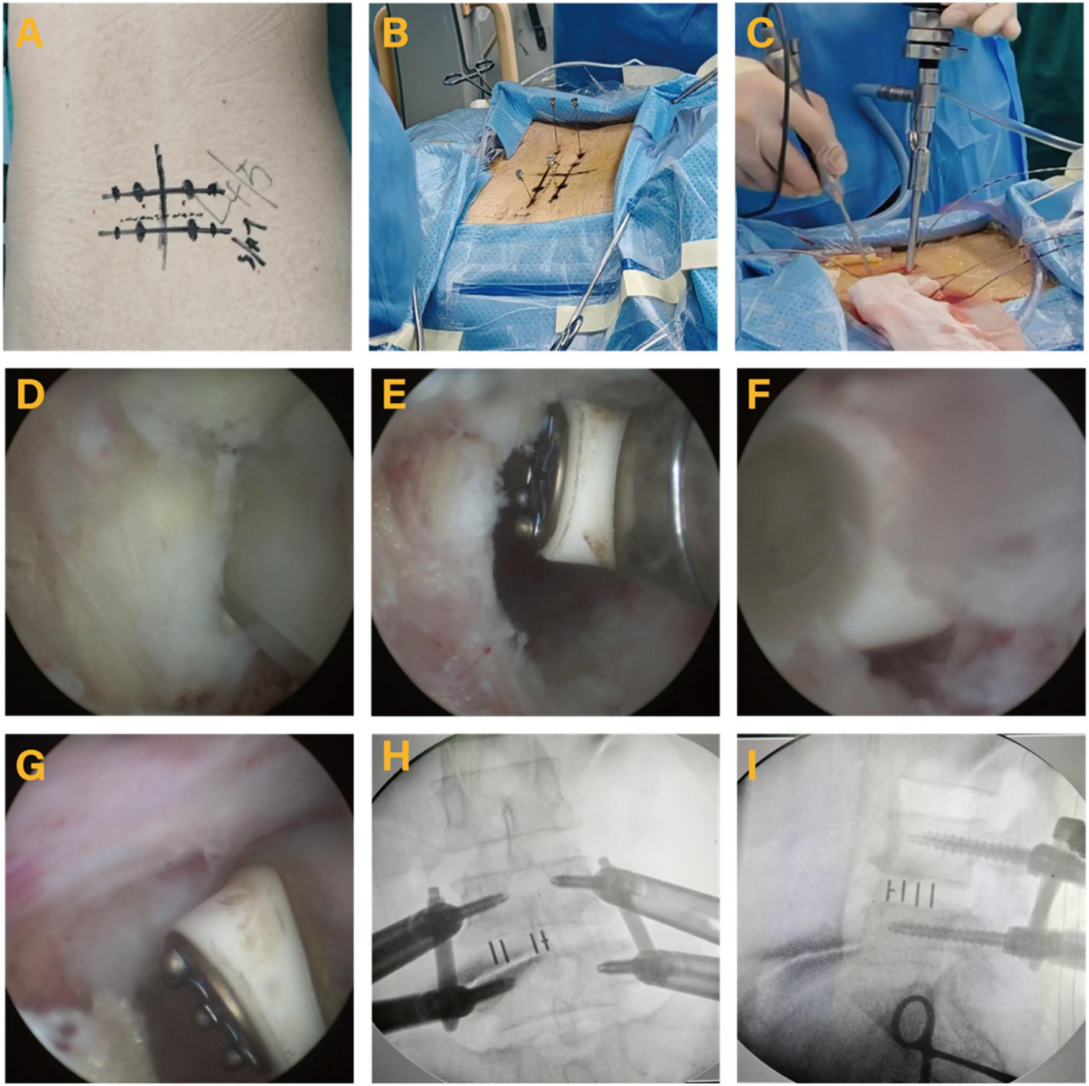
Unilateral biportal endoscopy lumbar interbody fusion (ULIF) surgical procedures. **(A)** Mark the lesion segment. **(B)** C-arm fluoroscopy to reconfirm surgical site. **(C)** Surgical site. **(D)** Intervertebral space confirmation. **(E)** Preparation of intervertebral space is completed. **(F)** A cage is meticulously positioned under endoscopic guidance. **(G)** The endoscopic view reveals the neural structures relaxed following decompression. **(H,I)** Images of final internal fixation.

**ULIF**: Under general anesthesia, the patient was positioned in the prone posture. C-arm fluoroscopy was utilized to identify and mark the surgical level and bilateral pedicles. After routine sterilization, guide needles were placed along each pedicle under C-arm fluoroscopy, and transverse incisions were made at the upper and lower pedicle markings on the more symptomatic side. Taking the right approach as an example, the upper incision serves as the operation channel, and the caudal incision serves as the observation channel, with the incision length of about 1.5 cm. Sequential dilators were inserted to establish the observation and operation channels. After connecting the light source, an arthroscope was introduced into the observation channel, and surrounding soft tissues were excised via the operative channel, with radiofrequency coagulation applied to control bleeding. The lower edge of the upper vertebra and the upper edge of the lower vertebra were exposed. Osteotomes were used to remove portions of the vertebral plate and medial articular eminence of the superior vertebra, along with parts of the ligamentum flavum, thereby exposing the dural sac and nerve root. The nerve root was retracted using a nerve retractor, and tissues such as the disc and nucleus pulposus were removed using a forceps and reamer to prepare the endplates. The endplate cartilage was verified as removed via endoscopic examination, and a suitably sized Polyetheretherketone (PEEK) cage was selected and filled with allograft bone. Autograft and allograft bone particles were packed around the cage. Spinal canal decompression was reassessed to ensure adequate relief. Finally, pedicle screws were placed along the guide needles under fluoroscopy, and the wound was closed after the drainage tube was positioned (Figure 1).

**Figure 2:**
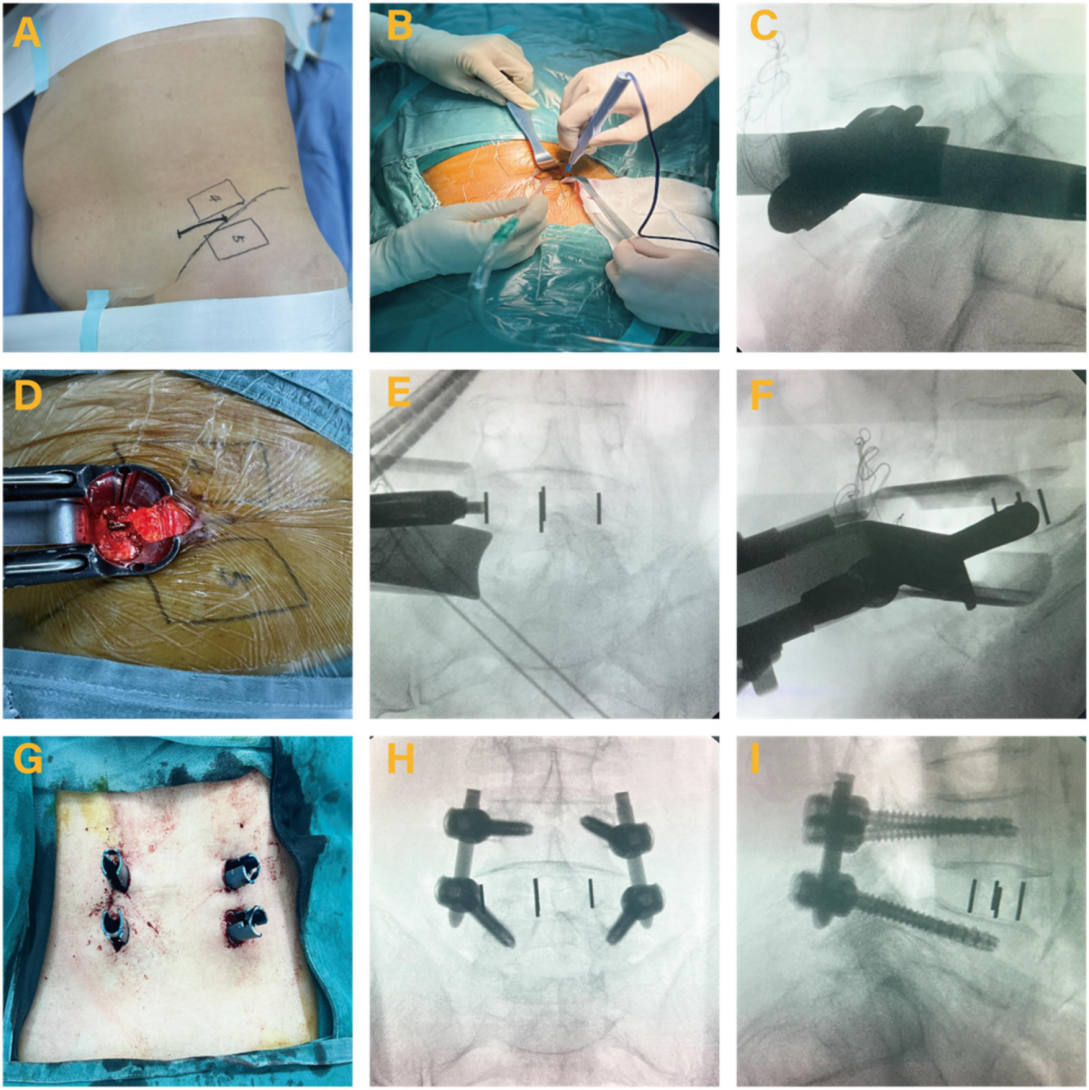
Oblique lumbar interbody fusion with posterior percutaneous screw fixation (OLIF-PF) surgical procedures. **(A)** Mark the lesion segment. **(B)** Surgical site. **(C)** C-arm fluoroscopy to reconfirm surgical site. **(D)** Preparation of intervertebral space is completed. **(E,F)** A cage is meticulously positioned. **(G)** Percutaneous pedicle screw placement is completed. **(H,I)** Images of final internal fixation.

**OLIF-PF**: After general anesthesia, the patient was positioned in the right lateral decubitus position with the back perpendicular to the operating table. The waist was supported with an arc cushion to align the surgical segments horizontally. C-arm fluoroscopy was used to identify and mark the surgical level. A 4 cm longitudinal incision was made at the midpoint of the segment. The skin, subcutaneous tissue, and fascia were sequentially incised, and the oblique abdominal muscles were bluntly separated along their fibers. The peritoneum and abdominal organs were retracted to expose the vertebral body, and a tubular retractor was inserted. C-arm fluoroscopy was used again to confirm the surgical level. After connecting the light source, the intervertebral disc and nucleus pulposus were removed to prepare the endplates. Test molds were used to determine cage size, followed by insertion of a PEEK cage filled with allograft bone. After confirming proper cage placement via fluoroscopy, the surgical area was irrigated, the wound was closed, and a drainage tube was placed. The patient was repositioned prone, and posterior pedicle screws and rods were inserted, with final screw placement confirmed using fluoroscopy (Figure 2).

### Clinical indicators

Demographic and clinical data, including sex, age, body mass index (BMI), diabetes, hypertension, course of disease, diagnosis, operation time (From the initial skin incision to the final closure of the wound), intraoperative blood loss, postoperative hospital stay, follow-up time, and complications, were collected for analysis. Visual analogue scale (VAS) and the Oswestry disability index (ODI) scores were used to assess the improvement of pain and function of the patients, which were assessed at 1 day preoperatively, 1 day postoperatively, 1, 3, 6, 12 months postoperatively, and at the last follow-up, respectively. All patients were followed up for more than 12 months. MacNab scale scores were used to assess pain relief at the last follow-up. Improvements can be classified into 4 grades: 75% to 100% (excellent, able to work normally), 50% to 74% (good, able to work less), 25% to 49% (fair, unable to work), 0% to 24% (poor, another surgery may be needed).

### Laboratory indicators

CRP and ESR were used to assess the level of inflammation in both groups, and Hb was used to assess the hemoglobin loss in both groups, which was measured at 1 day preoperatively and 1 day postoperatively.

### Radiological evaluation indicators

Lumbar x-ray and CT scans were performed preoperatively, 3 days postoperatively, at 12 months, and at the final follow-up. Lumbar lordosis (LL) and segmental angle (SA) were measured on lateral radiographs. Disc height (DH) was measured using CT midsagittal images, with the height calculated as the average of the anterior and posterior disc edges. Bone fusion was defined as the presence of continuous bridging trabecular bone between the cephalic and caudal endplates on both coronal and sagittal CT views, assessed at the last follow-up (17). Cage subsidence was classified according to the grading system reported by Marchi (18), which was based on the severity of height loss between the cage and the vertebral endplates: grade 0 = 0 to 24%, grade I = 25 to 49%, grade II = 50 to 74%, and grade III = 75% to total collapse of the level. Grades 0 and I were classified as low-grade subsidence, while grades II and III were considered high-grade.

### Statistical analysis

Statistical analysis was performed using SPSS 26.0 (IBM SPSS Statistics, Armonk, New York, USA). Continuous variables with normal distribution (e.g., age, BMI, disease duration, operation time, intraoperative blood loss, hospital stay, VAS scores, ODI, CRP, ESR, Hb, Hb changes, DH, LL, and SA) were presented as mean ± standard deviation and compared using the Student's t-test. Non-normally distributed continuous variables were analyzed using the Mann–Whitney U test. Categorical data (e.g., sex, diabetes, hypertension, diagnosis, surgical level, Modified MacNab score, and complications) were expressed as frequency or percentage and compared using the Chi-square test or Fisher's exact test. A *P*-value of <0.05 was considered statistically significant.

## Results

### Demographic data

A total of 96 patients (46 males and 50 females) were included, with 54 in the ULIF group and 42 in the OLIF-PF group. No significant differences were found between the groups in terms of sex, age, BMI, diabetes, hypertension, disease duration, diagnosis, surgical level, or follow-up time (*P* > 0.05, Table 1).

**Table 1:** Baseline characteristics and surgical-related outcomes of the included patients.

Characteristic	ULIF (*n* = 54)	OLIF-PF (*n* = 42)	*χ*^2^/t	*P*-value
Sex (male/female, n)	26/28	20/22	0.959	
Age (years)	65.54 ± 5.45	65.45 ± 6.09		0.408
BMI (kg/m^2^)	23.48 ± 2.52	23.10 ± 2.11		0.404
Diabetes (yes/no)	14/40	10/32	0.812	
Hypertension (yes/no)	13/41	12/30	0.618	
Course of disease (months)	21.28 ± 23.21	20.31 ± 21.91		0.579
Surgical level (n)			0.073	
L3/4	8	1		
L4/5	28	29		
L5/S1	18	12		
Follow-up time (months)	15.17 ± 3.77	14.88 ± 4.11		0.650
Operation time (mins)	132.72 ± 10.58	168.64 ± 14.12		**0.021**
Intraoperative blood loss (mL)	135.33 ± 26.46	93.90 ± 18.84		**0.023**
Hospital stay (days)	7.96 ± 1.67	7.33 ± 1.60		0.717
Modified MacNab score			0.823	
Excellent	39	30		
Good	10	8		
Fair	4	2		
Poor	1	2		

*χ*^2^, chi-square value; ULIF, unilateral biportal endoscopic transforaminal lumbar interbody fusion; OLIF-PF, oblique lumbar interbody fusion with posterior percutaneous screw fixation.

Data presented as mean ± standard deviation. Significant values (*P* < 0.05) are in bold.

### Surgical-related results

The ULIF group had a shorter operative time compared to the OLIF-PF group (ULIF: 132.72 ± 10.58; OLIF-PF: 168.64 ± 14.12; *P* < 0.05), but experienced greater intraoperative blood loss (ULIF: 135.33 ± 26.46; OLIF-PF: 93.90 ± 18.84; *P* < 0.05). No significant difference in hospital stay was observed between the two groups (ULIF: 7.96 ± 1.67; OLIF-PF: 7.33 ± 1.60; *P* = 0.717, Table 1).

### Clinical results

**Figure 3:**
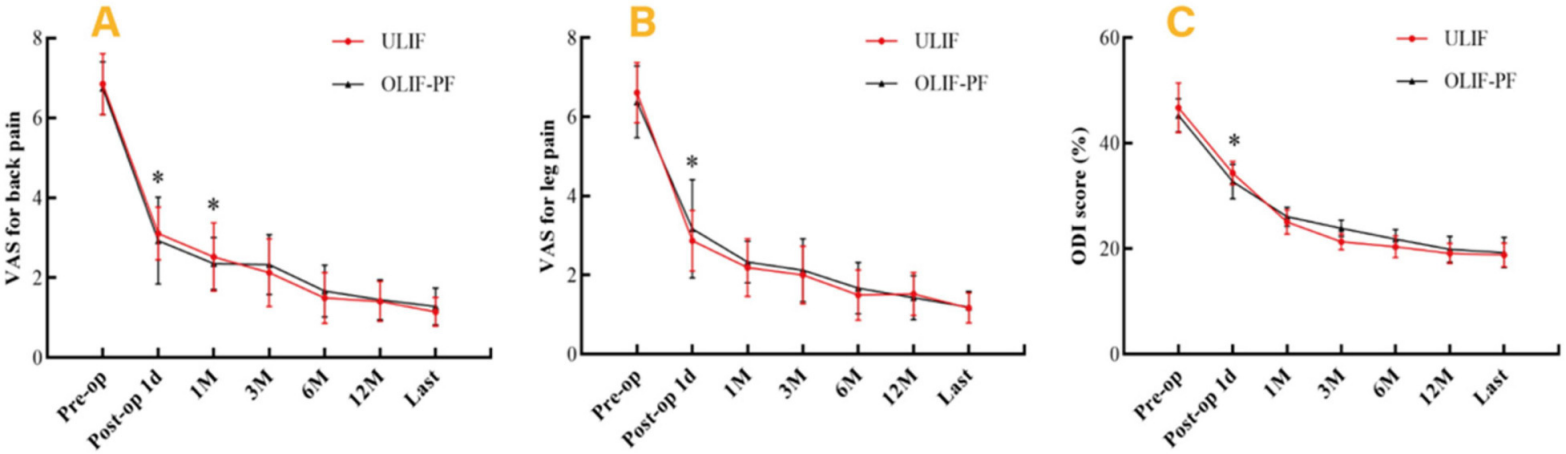
Comparison in clinical outcomes between the two groups at each follow-up time. **(A)** VAS for back pain, **(B)** VAS for leg pain, and **(C)** ODI. *P < 0.05 for the comparison between the two groups.

Preoperative VAS scores for leg and back pain and ODI scores were comparable between the groups (all *P* > 0.05). Postoperatively, both groups showed significant improvements in VAS and ODI scores for leg and back pain (all *P* < 0.05). At 1 day and 1 month postoperatively, the OLIF-PF group had significantly lower back pain VAS scores than the ULIF group (*P* = 0.000, *P* = 0.033, respectively). No significant differences were observed at 3, 6, or 12 months post-surgery. However, at the final follow-up, the ULIF group had significantly lower back pain VAS scores than the OLIF-PF group (*P* = 0.001). At 1 day postoperatively, the ULIF group had significantly lower leg pain VAS scores compared to the OLIF-PF group (*P* = 0.000), with no significant differences at other follow-up times. The ODI score at 1 day postoperatively was significantly lower in the OLIF-PF group (*P* = 0.000), with no significant differences between the groups at later follow-ups (Figures 3A–C, Table 2). At the last follow-up, the modified MacNab score in the ULIF group was excellent in 39 patients, good in 10 patients, fair in 4 patients, and poor in 1 patient, and the MacNab score in the OLIF-PF group was excellent in 30 patients, good in 8 patients, fair in 2 patients, and poor in 2 patients. There was no statistically significant difference between the two groups in the modified MacNab score (*P* = 0.823, Table 1).

**Table 2:** Comparison of VAS and ODI scores between the two groups at each follow-up time.

Variables	ULIF (*n* = 54)	OLIF-PF (*n* = 42)	*P*-value
VAS for back pain			
Preoperation	6.85 ± 0.76	6.74 ± 0.66	0.616
Postoperation 1 day^a^	3.11 ± 0.66	2.93 ± 1.09	**0.000**
Postoperation 1 month^a^	2.52 ± 0.88	2.36 ± 0.66	**0.033**
Postoperation 3 months^a^	2.13 ± 0.85	2.33 ± 0.75	0.292
Postoperation 6 months^a^	1.50 ± 0.64	1.67 ± 0.65	0.963
Postoperation 12 months^a^	1.41 ± 0.50	1.45 ± 0.50	0.421
Last follow up^a^	1.15 ± 0.36	1.29 ± 0.46	**0.001**
VAS for leg pain			
Preoperation	6.61 ± 0.76	6.38 ± 0.91	0.163
Postoperation 1 day^a^	2.87 ± 0.78	3.17 ± 1.12	**0.000**
Postoperation 1 month^a^	2.19 ± 0.73	2.33 ± 0.53	0.064
Postoperation 3 months^a^	2.00 ± 0.73	2.12 ± 0.80	0.255
Postoperation 6 months^a^	1.50 ± 0.64	1.67 ± 0.65	0.963
Postoperation 12 months^a^	1.52 ± 0.54	1.43 ± 0.55	0.797
Last follow up^a^	1.20 ± 0.41	1.17 ± 0.38	0.358
ODI (%)			
Preoperation	46.74 ± 4.14	45.26 ± 3.16	0.115
Postoperation 1 day^a^	34.31 ± 2.20	32.71 ± 3.26	**0.000**
Postoperation 1 month^a^	26.06 ± 2.30	26.05 ± 1.83	0.526
Postoperation 3 months^a^	21.33 ± 1.54	23.88 ± 1.55	0.721
Postoperation 6 months^a^	20.39 ± 2.06	21.74 ± 1.98	0.245
Postoperation 12 months^a^	19.11 ± 1.93	19.90 ± 2.45	0.066
Last follow up^a^	18.80 ± 2.25	19.50 ± 2.66	0.103

VAS, visual analogue scale; ODI, Oswestry disability index.

Data presented as mean ± standard deviation. The *P*-value indicates the comparison between two groups. Significant values (*P* < 0.05) are in bold.

^a^ Significantly different compared to preoperation.

### Inflammation-related indicators

No significant differences in CRP, ESR, or Hb levels were observed preoperatively between the two groups (all *P* > 0.05). The postoperative CRP and ESR levels were significantly higher compared to preoperative levels (*P* < 0.05), while there was no significant difference between the two groups postoperatively (*P* = 0.223, *P* = 0.272, respectively, Table 3).

**Table 3:** Comparison of the CRP, ESR and Hb between the two groups.

Variables	ULIF (*n* = 54)	OLIF-PF (*n* = 42)	*P*-value
CRP (mg/L)			
Preoperation	5.73 ± 2.11	5.70 ± 2.92	0.267
Postoperation^a^	25.48 ± 10.58	24.83 ± 14.37	0.223
ESR (mm/L)			
Preoperation	9.69 ± 1.24	9.26 ± 1.06	0.754
Postoperation^a^	22.30 ± 1.50	21.75 ± 1.08	0.272
Hb (g/L)			
Preoperation	128.93 ± 12.05	125.36 ± 9. 90	0.107
Postoperation^a^	117.54 ± 10.83	116.36 ± 10.37	0.556
Hb changes	11.39 ± 6.86	9.02 ± 3.49	**0.013**

CRP, C-reaction protein; ESR, erythrocyte sedimentation rate; Hb, hemoglobin.

Preoperation defined as 1 day before operation; Postoperation defined as 1 day after operation. Data presented as mean ± standard deviation. The *P*-value indicates the comparison between two groups. Significant values (*P* < 0.05) are in bold.

^a^ Significantly different compared to preoperation.

Postoperative Hb levels decreased significantly in both groups (*P* < 0.05), with no significant difference between the groups (*P* = 0.556). However, the Hb changes in the OLIF-PF group were significantly lower than in the ULIF group (*P* = 0.013, Table 3).

### Radiological results

Preoperative DH, LL, and SA values were comparable between the groups (all *P* > 0.05). Both groups showed significant improvements in DH, LL, and SA at 3 days postoperatively (*P* < 0.05). However, the OLIF-PF group exhibited superior improvements in DH, LL, and SA at 3 days postoperatively (*P* = 0.025, *P* = 0.024, *P* = 0.029, respectively, Table 4). At 12 months and at the final follow-up, both groups maintained favorable improvements in DH, LL, and SA compared to preoperative values (*P* < 0.05), with no significant differences between the groups (all *P* > 0.05, Table 4). At the final follow-up, 52 patients (96.3%) in the ULIF group and 39 patients (92.9%) in the OLIF-PF group achieved interbody bony fusion, with no significant difference in fusion rates between the groups (*P* = 0.772, Table 4).

**Table 4:** Comparison of the radiographic results between the two groups.

Variables	ULIF (*n* = 54)	OLIF-PF (*n* = 42)	*P*-value
DH (mm)			
Preoperation	8.53 ± 0.65	8.77 ± 0.60	0.586
Postoperation^a^	12.62 ± 1.44	13.82 ± 0.98	**0.025**
Postoperation 12 months^a^	12.53 ± 1.46	12.92 ± 1.73	0.151
Last follow up^a^	12.36 ± 1.47	12.34 ± 1.84	0.099
LL (°)			
Preoperation	36.89 ± 4.07	37.43 ± 3.33	0.432
Postoperation^a^	42.29 ± 3.62	43.20 ± 1.61	**0.014**
Postoperation 12 months^a^	41.89 ± 3.15	41.85 ± 2.11	0.161
Last follow up^a^	41.47 ± 3.35	41.11 ± 2.02	0.074
SA (°)			
Preoperation	8.34 ± 1.11	8.52 ± 1.34	0.392
Postoperation^a^	12.71 ± 2.03	13.79 ± 1.41	**0.029**
Postoperation 12 months^a^	12.52 ± 1.65	12.96 ± 1.90	0.464
Last follow up^a^	12.24 ± 1.77	12.48 ± 1.90	0.617
Fusion rate at last follow-up (%, n)	96.30% (52)	92.9% (39)	0.772

DH, disc height; LL, lumbar lordosis; SA, segmental angle; n, number of patients.

Preoperation defined as 1 day before operation; Postoperation defined as 3 days after operation. Data presented as mean ± standard deviation. The *P* value indicates the comparison between two groups. Significant values (*P* < 0.05) are in bold.

^^a^^ Significantly different compared to preoperation.

### Complications

There were four cases of complications in the ULIF group, with a complication rate of 7.4% (4/54). One case had an intraoperative dural tear, and we compressed the rupture with a gelatin sponge, without detecting any cerebrospinal fluid leakage postoperatively. One case had an epidural hematoma detected on postoperative Magnetic Resonance Imaging (MRI), with no intervention, as it was not accompanied by any clinical symptoms. Two cases presented with cage subsidence at the last follow-up, both of which were grade I, and no intervention was required, as it was not accompanied by any clinical symptoms. There were six cases of complications in the OLIF-PF group, with a complication rate of 14.3% (6/42). One patient sustained a left sympathetic nerve trunk injury, which resolved after 3 months of functional exercise and nutrition therapy. One case had transient lateral thigh numbness, which had improved after conservative treatment. Four cases had cage subsidences at the last follow-up, three of which were grade I and one was grade II, all of which were not accompanied by clinical symptoms and required no treatment. No significant difference in complication rates was found between the two groups (*P* = 0.449, Table 5).

**Table 5:** Comparison of the complications between the two groups.

Variables	ULIF (*n* = 54)	OLIF-PF (*n* = 42)	*P*-value
Total (%, n)	7.4% (4)	14.3% (6)	0.449
Dura tear	1.9% (1)	0	
Epidural hematoma	1.9% (1)	0	
Nerve injury	0	2.4% (1)	
Transient thigh numbness	0	2.4% (1)	
Cage subsidence	3.7% (2)	9.5% (4)	0.457

## Discussion

The growing emphasis on rapid rehabilitation has driven the advancement of minimally invasive spine techniques. Spine surgeons are increasingly focused on exploring minimally invasive approaches to reduce complications associated with traditional open LIF procedures. Minimally invasive LIF and endoscopic-assisted LIF are currently receiving significant attention and recognition from spine surgeons. Compared to traditional TLIF surgery, OLIF possesses advantages in minimizing surgical injury, reducing blood loss, and reconstructing spinal sequences (19). OLIF allows greater cage to enter and achieves indirect decompression of the spinal canal by restoring the intervertebral height. OLIF-PF combines posterior pedicle screw fixation with OLIF to reduce the incidence of postoperative cage subsidence after OLIF alone (1), compensating for the deficiencies of OLIF alone. Notably, in patients with severe central spinal stenosis, the OLIF approach is contraindicated because it is difficult to achieve adequate decompression of the spinal canal, which will result in persistent stenosis symptoms. Studies (20) have shown that severe central stenosis is a major factor influencing the need for subsequent direct posterior decompression after OLIF. And compared to conventional LIF, ULIF is associated with less injury, lower inflammation, less bleeding, and faster postoperative recovery (2, 21). Since Heo first (22) combined UBE technology with TLIF in 2017, the indications for ULIF have expanded to include a broader range of lumbar degenerative conditions.

Perioperative indicators are gradually coming into focus. Our study found that OLIF-PF had a longer operation time compared to ULIF, as patients in the OLIF-PF group needed to be repositioned from lateral to prone. However, blood loss was significantly lower in the OLIF-PF group, consistent with findings from previous studies (23). ULIF requires osteotomy of the vertebral plate and upper and lower articular processes to create access for decompression and cage placement, bleeding on the surface of the bone after osteotomy, and from the rupture of the spinal venous plexus is unavoidable (24), which may be contributing to more bleeding in ULIF. Nevertheless, ULIF still has advantages over conventional lumbar fusion in reducing blood loss (21).

Our study showed significant improvements in VAS scores for leg and back pain, as well as ODI scores, in both groups postoperatively, with improvements maintained through the final follow-up, consistent with previous studies (25, 26). Notably, the OLIF-PF group had significantly lower back pain VAS scores than the ULIF group at 1 day and 1 month postoperatively. At the final follow-up, the ULIF group showed significantly better VAS scores for back pain than the OLIF-PF group. This may be due to OLIF-PF's avoidance of paraspinal muscle dissection and laminectomy, which are required in ULIF (10). Additionally, the severity of stenosis and its location may also be significant factors influencing the VAS for back pain. ULIF, by providing direct decompression to the posterior spinal structures, may effectively mitigate the impact of severe stenosis, resulting in improved VAS for back pain at the final follow-up.

This explains the additional advantage of OLIF-PF in reducing back pain postoperatively, and the same difference was demonstrated between the ODI scores of the two groups postoperatively. In addition, sagittal spinopelvic alignment is closely associated with low back pain following spinal surgery (27). Previous studies have demonstrated that OLIF effectively restores patients' sagittal spinopelvic alignment, thereby significantly alleviating low back pain symptoms (28).

Long-term outcomes for low back pain after OLIF were less promising in our study. OLIF utilizes larger cages to distract DH, reduce disc bulging, and elongate the hypertrophied ligamentum, indirectly decompressing neural structures. However, Kaliya's study identified increased DH as a risk factor for intervertebral space loss after initial distraction (29). Larger cage sizes indicate excessive axial stress, which may cause damage to the endplates, especially the upper endplates (30). We hypothesize that the recurrence of symptoms after OLIF-PF is due to significant loss of intervertebral height from cage subsidence, which in turn increases strain on the nerve roots (29). However, high-quality studies are still needed in the future to demonstrate the association between higher DH and loss of intervertebral height after OLIF-PF.

VAS for leg after surgery was better in the ULIF group than in the OLIF-PF, while there was no significant difference in VAS for leg between the two groups at other follow-up times. Similar to TLIF, ULIF can directly remove the thickened ligamentum flavum and intervertebral discs intraoperatively to achieve direct decompression of the spinal canal. Zhao et al. (10) demonstrated that TLIF has the advantage of enlarging the cross-sectional area of the spinal canal and provides better decompression compared to OLIF. In our study, we also found that ULIF had a better decompression effect in the short-term follow-up time, and in the long-term follow-up time, both groups showed satisfactory outcomes.

Rehabilitation of sagittal balance after lumbar fusion surgery has a significant impact on patient prognosis (31). Champagne et al. showed (32) that OLIF provided the optimal outcomes in restoring lumbar DH, LL, and SA when compared to TLIF. Similar outcomes were observed in our study, with better improvement in DH, LL, and SA in the OLIF-PF group than in the ULIF group postoperatively. This is not surprising, given that OLIF was the only approach to significantly improve segmental and overall lumbar lordosis, as well as being the approach with the highest gain in regard to disk height (32). OLIF provides easier access to the anterior column spine, and a larger cage can partially explain such results. CRP and ESR reflect the inflammation level of the body, and our study showed that there was no significant difference between the two groups in inflammation levels, suggesting that, benefiting from the minimally invasive surgical approaches, the two groups had comparable outcomes in damage control. There was no difference between the two groups in postoperative Hb; however, the Hb changes were greater in the ULIF group, which is consistent with our observation of more blood loss in ULIF. At the last follow-up, 52 cases (96.3%) of the ULIF group and 39 cases (92.9%) of the OLIF-PF group had reached interbody bony fusion, and there was no significant difference in fusion rates between the two groups. A recent Meta-analysis showed (33) that the fusion rate after OLIF was about 90.75%. And the fusion rate after ULIF is more than 90% (34), which is similar to that of MI-TLIF. Our study showed similar outcomes, indicating that satisfactory fusion results were reached with both fusion modalities. When calculating the complication rates of the two groups, no differences were found between the two groups in the complication rates, and no serious outcomes were observed in either group. Both groups of patients presented with cases of cage subsidence but without showing corresponding clinical symptoms. Previous studies have shown that patients with osteoporosis have a higher probability of cage subsidence (35, 36), and excessive spreading of the intervertebral space during surgery will also contribute to the interbody height loss during the early follow-up process (37). Therefore, we recommend routine measurement of BMD preoperatively, and cautious intraoperative use of larger cages will help to avoid fusion subsidence. It is noteworthy that although cage subsidence showed no significant difference between the two groups, the ULIF technique resulted in slightly less cage subsidence. ULIF allows for endplate manipulation under magnified visualization, providing a more optimal fusion environment and positioning for the cage (38). This potential technical advantage may explain the results of our study.

This study has some limitations that should be acknowledged. It was a single-center, retrospective cohort study with a relatively small sample size, and some potential differences between the two groups may not have been detected. Larger samples and more institutional participation are needed in the future to validate the differences between these two surgical modalities. In addition, there was selection bias as patients with severe central lumbar spinal stenosis were all assigned to the ULIF group after being informed of the surgery. This may lead to the emergence of selection bias, although such bias stems from the indications for surgery and the premise of ensuring patient safety. Future research focused on degenerative lumbar diseases is needed to further confirm the clinical efficacy of both approaches. Furthermore, the follow-up period in this study was relatively short, and longer follow-up durations will be necessary to establish the long-term clinical effects.

## Conclusion

Both ULIF and OLIF-PF demonstrated positive mid-term outcomes for the treatment of LSCS, with each surgical technique showing effectiveness. Both methods significantly improved pain levels and restored lumbar sagittal alignment. Nevertheless, surgical strategies should be tailored to each patient's specific clinical presentation and the surgical indications.

## Data Availability

The original contributions presented in the study are included in the article/Supplementary Material, further inquiries can be directed to the corresponding author.
